# A Case Series on Pregnant Patients with Mild Covid-19 Infection and Signs of Severe Placental Insufficiency

**DOI:** 10.1155/2023/2018551

**Published:** 2023-03-28

**Authors:** A. Ivert, C. Lindblad Wollmann, K. Pettersson

**Affiliations:** Department of Obstetrics and Gynecology, Karolinska University Hospital, Stockholm, Sweden

## Abstract

In this case series, we present five cases of pregnant women who sought medical attention for reduced fetal movements with an ongoing mild maternal Covid-19 infection at a Stockholm hospital in Spring of 2021. At the time of admission, the patients were in gestational week between 24 + 0 and 33 + 5. Abdominal ultrasound at the hospital showed no fetal movements, and cardiotocography (CTG) was pathological. All women delivered via cesarean section within 24 hours after admission. Placental pathology in all cases showed massive perivillous fibrin deposition and extensive histiocytic intervillositis. All placentas were Covid-19 polymerase chain reaction (PCR) positive. The infants were Covid-19 PCR negative. Consistent with other published case reports, we hypothesize that Severe acute respiratory syndrome coronavirus 2 (SARS-CoV-2) can affect the placenta resulting in massive perivillous fibrin deposition and histiocytic intervillositis leading to acute placental insufficiency and fetal hypoxia. The absence of intrauterine growth restriction also augments the theory of an acute onset of placental insufficiency due to the Covid-19 infection.

## 1. Introduction

One of many questions at the beginning of the Covid-19 pandemic was the potential risk and specific effects of Covid-19 on pregnant women and their unborn children.

Since the start of pandemic, many studies have set out to better understand SARS-CoV-2 effect on pregnancy. More than 2 years into the pandemic, it is a well-known fact that the infection can affect pregnant women giving them a significantly increased risk of maternal mortality and morbidity [1]. Several studies have concluded that pregnant women have a higher risk for more severe diseases than non-pregnant women [2]. Although Covid-19 most often presents as a respiratory tract infection, several organ systems can be affected [3].

Preeclampsia, fetal distress, low birth weight, and reduced fetal growth are well known to be related to placental dysfunction [4]. The Intercovid group showed that a Covid-19 infection during pregnancy increases the risk of these conditions [1, 5]. They also showed that SARS-CoV-2 infected women with diabetes have an increased risk of severe disease compared to healthy mothers [6].

A few studies and case reports have described placental complications and transplacental transmission of Covid-19 [7, 8].

Zaigham et al. described a case of transplacental transmission with fibrinoid depositions of up to 50% of the placental volume [9]. Another case report describes a Covid-19 infected pregnant woman with severe preeclampsia and placental abruption, where pathology of the placenta showed presence of diffuse perivillous fibrin and an inflammatory infiltrate composed of macrophages as well as histiocytic intervillositis. The placenta was positive for SARS-CoV-2 by polymerase chain reaction (PCR); the infant, however, tested negative [10]. Marton et al. described a SARS-CoV-2 positive pregnant woman with reduced fetal movements. Ultrasound showed intrauterine fetal death and placental pathology with massive perivillous fibrin deposition (MPVFD), histiocytic intervillositis, and was SARS-CoV-2 positive [11].

Previous studies show that MPVFD has been associated with an increased risk of intrauterine growth restriction, preterm birth, and intrauterine fetal death (IUFD). MPVFD has also been described to correlate to maternal autoimmune disease and some viral infections [12–15].

During the third wave of Covid-19 in Sweden, we noticed some cases of pregnant women with an ongoing mild Covid-19 infection who sought medical care for reduced fetal movement with the consequence of delivery due to signs of fetal asphyxia within 24 hours after admission. Placental pathology in these cases showed MPVFD and extensive histiocytic intervillositis. Together with macroscopic and microscopic examination of the placenta at the Center for Perinatal Pathology at Karolinska, the placentas were analyzed for SARS-CoV-2 using immunohistochemical stain for protein ORF3A as well as molecular analysis (mRNA COVN and COVS) [16].

## 2. Case 1

A nulliparous 30-year-old woman of European ethnicity presented with reduced fetal movements for 1 day in gestational week 24 + 4. She had a body mass index (BMI) of 26 kg/m^2^ and was generally healthy. The previous days she had experienced light cold symptoms and had tested PCR positive for SARS-CoV-2 in a combined nasopharynx (NPH) throat swab.

**Figure 1:**
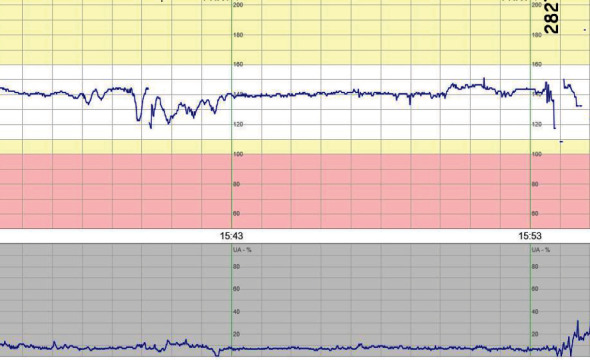
Patient 1 CTG at admittance to the hospital.

Upon admittance to the obstetric unit, the ultrasound showed no fetal movements, normal heart activity, and normal amount of amniotic fluid. The fetus had absent/reversed end-diastolic umbilical artery blood flow (blood flow class [BFC] 3a–3b). Cardiotocography (CTG) was interpreted as pathological (Figure 1). A few hours later CTG had not improved, and the decision was made to deliver with cesarean section. A boy was delivered weighing 660 g and with an Apgar score of 7, 8, and 8 at 1, 5, and 10 minutes, respectively. The umbilical cord gases showed mild asphyxia with an arterial pH of 7.21 and base excess (BE) −9. The infant was admitted to the neonatal intensive care unit (NICU) and subsequently tested negative for SARS-CoV-2 in NPH 12 hours postpartum.

Placental pathology showed MPVFD with approximately 80% of the placenta affected, and extensive histiocytic intervillositis was reported. Immunohistochemistry and molecular analysis for SARS-CoV-2 were positive. Placenta weight 166 g.

## 3. Case 2

A nulliparous 31-year-old woman of European ethnicity presented with complete loss of fetal movements for 4 days in gestational week 31 + 4. She had a BMI of 22 kg/m^2^ and was generally healthy. In the previous days, she had experienced fever and light dyspnea and had a PCR positive test for Covid-19 in a combined NPH throat swab 8 hours postpartum.

**Figure 2:**
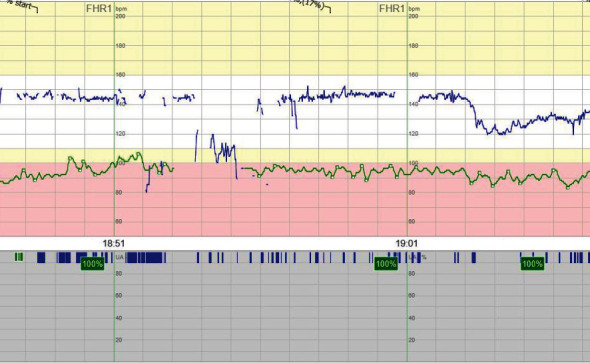
Patient 2 CTG at admittance to the hospital.

An ultrasound conducted at the obstetric emergency unit displayed no fetal movements but normal heart activity and normal amount of amniotic fluid. CTG was pathological (Figure 2). The decision to deliver by cesarean section was made, and a boy weighing 1662 g was delivered 4 hours after admittance to the hospital with an Apgar score of 3, 5, and 6 at 1, 5, and 10 minutes, respectively. The umbilical cord gases showed severe asphyxia, with an arterial pH of 6.95 and BE −18.5. The infant was admitted to NICU and later tested negative for SARS-CoV-2 in NPH.

Placental pathology showed MPVFD, approximately 50% of the placenta was affected, and extensive histiocytic intervillositis was reported. Immunohistochemistry and molecular analysis for SARS-CoV-2 were positive. Placenta weight 265 g.

## 4. Case 3

A nulliparous 30-year-old woman of Middle Eastern ethnicity presented with reduced fetal movements for 1 day in gestational week 26+4. Her medical history consisted of endometriosis but she was otherwise healthy. Her BMI was 19 kg/m^2^. In the previous 12 days, she had experienced light cold symptoms and had tested PCR positive for SARS-CoV-2 in a combined NPH throat swab.

**Figure 3:**
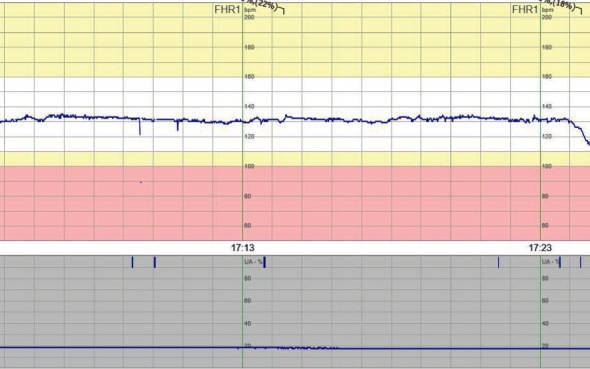
Patient 3 CTG at admittance to the hospital.

CTG upon admission was deemed preterminal (Figure 3), and an abdominal ultrasound showed no signs of fetal movements. The heart activity and the amount of amniotic fluid were normal. The fetus was growth restricted with absent/reversed end-diastolic umbilical artery blood flow (BFC 3a–3b). A girl was delivered through cesarean section with a weight of 686 g (small for gestational age) and an Apgar score of 3, 9, and 9 at 1, 5, and 10 minutes, respectively. The umbilical cord gases showed moderate asphyxia with an arterial pH of 7.02 and BE −18.2. The infant was admitted to NICU and later tested negative for SARS-CoV-2 in NPH and serum 2 days postpartum.

Placental pathology showed MPVFD, approximately 60% of the placenta was affected, and extensive histiocytic intervillositis was reported. Immunohistochemistry and molecular analysis for SARS-CoV-2 were positive. Placenta weight 197 g.

## 5. Case 4

A 3-gravida, 2-para 31-year-old woman of European ethnicity presented at the obstetric emergency unit with loss of fetal movements for 1 day in gestational week 24 + 0. She was healthy with a BMI of 35 kg/m^2^. In the previous days, she had experienced fever and a light headache, and she had tested PCR positive for SARS-CoV-2 in a combined NPH throat swab.

**Figure 4:**
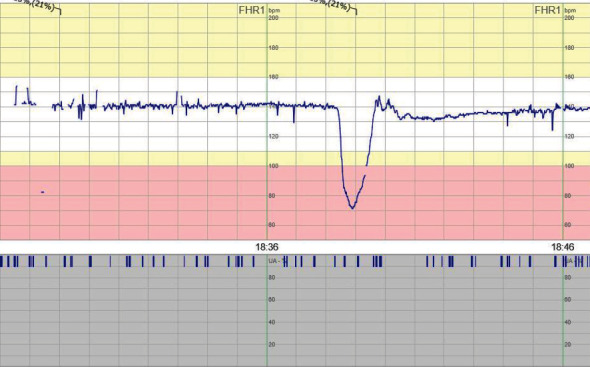
Patient 4 CTG at admittance to the hospital.

At admittance, CTG was deemed suspicious pathological, and the abdominal ultrasound was without fetal movements, normal heart activity, and amount of amniotic fluid. The flow in the umbilical cord was suspected pathological (BFC 1–2). The following day the CTG was pathologic (Figure 4), and the baby was delivered by cesarean section. A girl was born with a weight of 570 g and an Apgar score of 2, 5, and 7 at 1, 5, and 10 minutes, respectively. The umbilical cord gases showed moderate asphyxia with a venous pH of 7.07. The infant was admitted to NICU and later tested negative for SARS-CoV-2 in NPH and serum 16 hours postpartum. The child developed a severe intracranial hemorrhage and died at the age of 24 hours.

**Figure 5:**
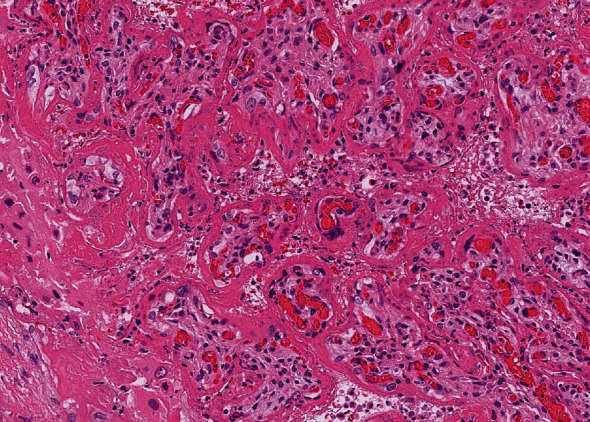
Microscopic picture descripting trophoblast necrosis, chronic histiocytic intervillositis, and perivillous fibrin deposition. Placenta from Case 4.

**Figure 6:**
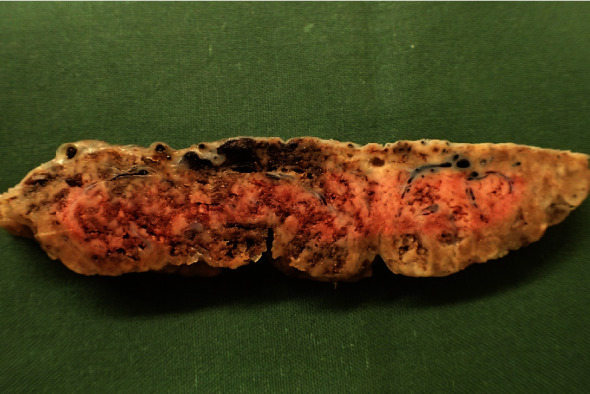
Placenta with sign of massive perivillous fibrin deposition. Placenta from Case 4.

Placental pathology showed MPVFD, approximately 90% of the placenta was affected, and extensive histiocytic intervillositis was reported (Figures 5 and 6). Immunohistochemistry and molecular analysis for SARS-CoV-2 were positive. Placenta weight 156 g. An autopsy was performed on the fetus and subsequently tested negative for SARS-CoV-2 in lung tissue.

## 6. Case 5

A nulliparous 41-year-old woman of European ethnicity presented with reduced fetal movements in gestational week 33+5. Her medical background consisted of pulmonary sarcoidosis and she had a BMI of 38 kg/m^2^. In the previous days, she had experienced light cold symptoms and tested PCR positive for SARS-CoV-2 in a combined NPH throat swab.

**Figure 7:**
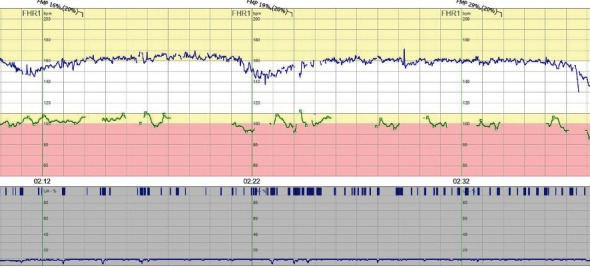
Patient 5 CTG at admittance to the hospital.

CTG upon admission was deemed pathological (Figure 7). An ultrasound showed no signs of fetal movement and oligohydramnios. The infant was delivered via an emergency cesarean section within 1 hour from admittance. A boy with a weight of 1780 g was delivered with an Apgar score of 3, 6, and 8 at 1, 5, and 10 minutes, respectively. The umbilical cord gases were normal, with an arterial pH of 7.22 and BE −6. SARS-CoV-2 PCR in NPH on the infant was negative 2 days postpartum.

Placental pathology showed MPVFD, approximately 85% of the placenta was affected, and extensive histiocytic intervillositis was described. Immunohistochemistry and molecular analysis for SARS-CoV-2 were positive. Placenta weight 382 g.

## 7. Discussion

This is a report of five pregnant women in gestation week 24–33 with mild Covid-19 symptoms who presented at an obstetric unit due to reduced fetal movements and signs of fetal asphyxia, where pathology analysis showed severe placental insufficiency (Table 1). None of the women were vaccinated against Covid-19. Four out of the five women were delivered via cesarean section within hours after admittance to the hospital due to pathological CTG at admission and no sign of fetal movements on abdominal ultrasound. Four out of the five women were treated with low molecular heparin after caesarian section and ongoing Covid-19 infection in accordance with Swedish guidelines [17].

All placentas had MPVFD, between 50% and 90% of the parenchyma was affected, and extensive histiocytic intervillositis was present. All placentas also tested positive for SARS-CoV-2. None of the infants tested positive for SARS-CoV-2. The low severity of the infection in the mother did not seem to correlate with the extensive placental findings and the effect of the infant in these cases.

Our findings are supported by previously published studies reporting on presence of placentitis as the main pathological finding during Covid-19 infection and pregnancy [18–20].

Schwartz et al. [18] looked at 68 placentas in cases where a Covid-19 infected mother suffered either stillbirth or early neonatal death and found presence of placental insufficiency due to MPVFD, chronic histiocytic intervillositis, and trophoblast necrosis. Watkins et al. [19] suggested a triad of placental findings of MPVFD, chronic histiocytic intervillositis, and trophoblast necrosis in SARS-CoV-2 infected women. Similarly, to our report, none of the neonates in their study tested positive for SARS-CoV-2.

Horn et al. [21] reported on two cases of intrauterine deaths in low symptomatic women, where placental pathology showed MPVFD and histiocytic intervillositis.

In a large case series, Stenton et al. [22] concluded that presence of SARS-CoV-2 placentitis is associated with increase of pregnancy loss. They did not see a correlation between severity of disease and placental findings.

Vaccination against Covid-19 had just started in Sweden in Spring of 2021 why none of the women descripted in this report had been immunized. The Intercovid group published a report in 2023 showing reduced risk for severe symptoms, complications, and death in women with complete or boosted vaccine doses [23].

We hypothesize that SARS-CoV-2 can affect the placenta resulting in MPVFD and histiocytic intervillositis leading to placental insufficiency and fetal hypoxia. The absence of intrauterine growth restriction suggests acute onset of placental insufficiency. However, this report is based on a case series with limited material to make any significant conclusion. Further studies are needed on placental findings and management of women with a Covid-19 infection. We recommend all pregnant women and women planning a pregnancy to get vaccinated to reduce the risk of complications.

## Figures and Tables

**Table 1:** Summary of all five cases with maternal characteristics, fetal outcome, and placental pathology.

Maternal characteristics					
Age (years)	30	31	30	30	41
Region of birth	European	European	Middle Eastern	European	European
BMI (kg/m^2^)	26	22	19	35	38
Obstetrical history∗	G1, P0	G1, P0	G1, P0	3G, P1	G1, P0
Co-morbidities	None	None	Endometriosis	BMI	Pulmonary sarcoidosis, BMI
Immunization Covid-19	No	No	No	No	No
Fetal outcome					
Gestational week at admission	24 + 4	31 + 4	26 + 4	24 + 0	33 + 5
Level of asphyxia	Mild	Severe	Moderate	Moderate	Normal
Infant NPH PCR Covid-19	Negative	Negative	Negative	Negative	Negative
Placenta pathology					
Amount of massive perivillous fibrin deposition and extensive histiocytic intervillositis					
	80	50	60	90	85
SARS-CoV-2 test					
	Positive	Positive	Positive	Positive	Positive

∗G = amount of pregnancies, P = amount of births.

## Data Availability

The data is collected from medical records and is not available for readers due to patient security.

## Abbreviations

BE: Base excess
BFC: Blood flow class
BMI: Body mass index
CTG: Cardiotocography
IUFD: Intrauterine fetal death
MPVFD: Massive perivillous fibrin deposition
NICU: Neonatal intensive care unit
NPH: Nasopharynx
PCR: Polymerase chain reaction.
